# What influences GPs’ use of pelvic examination? A qualitative investigation in primary care

**DOI:** 10.3399/BJGP.2022.0363

**Published:** 2023-04-18

**Authors:** Pauline Williams, Peter Murchie, Maggie E Cruickshank, Christine M Bond, Christopher D Burton

**Affiliations:** Aberdeen Centre for Women’s Health Research, Institute of Applied Health Sciences, University of Aberdeen, Aberdeen Maternity Hospital, Aberdeen.; Academic Primary Care, Institute of Applied Health Sciences, University of Aberdeen, Aberdeen.; Aberdeen Centre for Women’s Health Research, Institute of Applied Health Sciences, University of Aberdeen, Aberdeen Maternity Hospital, Aberdeen.; Academic Primary Care, Institute of Applied Health Sciences, University of Aberdeen, Aberdeen.; Academic Unit of Primary Care, University of Sheffield, Sheffield.

**Keywords:** clinical examination, gynaecological cancer, pelvic examination, primary health care, qualitative research, general practice

## Abstract

**Background:**

Omission of pelvic examination (PE) has been associated with diagnostic delay in women diagnosed with gynaecological cancer. However, PEs are often not carried out by GPs.

**Aim:**

To determine the perceptions of GPs about the role of PEs, the barriers to and facilitators of PEs, and GPs’ experience of PEs in practice.

**Design and setting:**

Qualitative semi-structured interview study conducted in one health board in Scotland (mixed urban and rural) with an approximate population of 500 000.

**Method:**

Interviews were conducted face-to-face or by telephone between March and June 2019. Framework analysis used the COM-B behaviour change model concepts of capability, opportunity, and motivation.

**Results:**

Data was compatible with all three domains of the COM-B framework. Capability related to training in and maintenance of skills. These went beyond carrying out the examination to interpreting it reliably. Opportunity related to the clinical environment and the provision of chaperones for intimate examination. Interviewees described a range of motivations towards or against PEs that were unrelated to either capability or opportunity. These all related to providing high-quality care, but this was defined in different ways: ‘doing what is best for the individual’, ‘doctors examine’, and ‘GPs as pragmatists’.

**Conclusion:**

GPs’ reasons for carrying out, or not carrying out, PEs in women with symptoms potentially indicating cancer are complex. The COM-B framework provides a way of understanding this complexity. Interventions to increase the use of PEs, and critics of its non-use, need to consider these multiple factors.

## INTRODUCTION

Clinical examination is traditionally viewed as an essential skill for a doctor.^1^ Pelvic examination (PE) consists of inspection of the vulva, bimanual examination of the pelvic organs, and visualisation of the cervix by speculum examination. The intimate nature of a PE can make it a challenging examination for both clinicians and patients but there is evidence that lack of a PE is associated with diagnostic delay.^2^^–^^4^ PE can provoke feelings of awkwardness, discomfort, embarrassment around getting undressed, and loss of dignity,^5^^–^^7^ engendering a feeling of vulnerability, and some women avoid PEs altogether.^8^

GPs have a professional duty to manage the intimacy of PEs as well as to ensure the examination is carried out when clinically required. This responsibility has been acknowledged by the General Medical Council (GMC), who have produced relevant guidance.^9^ One study has shown that GPs would carry out a pre-referral PE in only 37% of women who present with a gynaecological problem requiring referral to a specialist.^10^

PE requires dexterity and sensitivity; however, if a clinician does not carry out the examination frequently, the skills, or confidence in those skills, may decline along with the ability to distinguish normal from abnormal findings, and the willingness to undertake the procedure.^11^^,^^12^

Sensitive topics such as clinical behaviour can be explored more fully using qualitative methods. This qualitative study answered the question: ‘What are the perceptions and experiences of GPs on the role of primary-care-based PE, and the barriers to and facilitators of undertaking it?’

**Figure 1.**
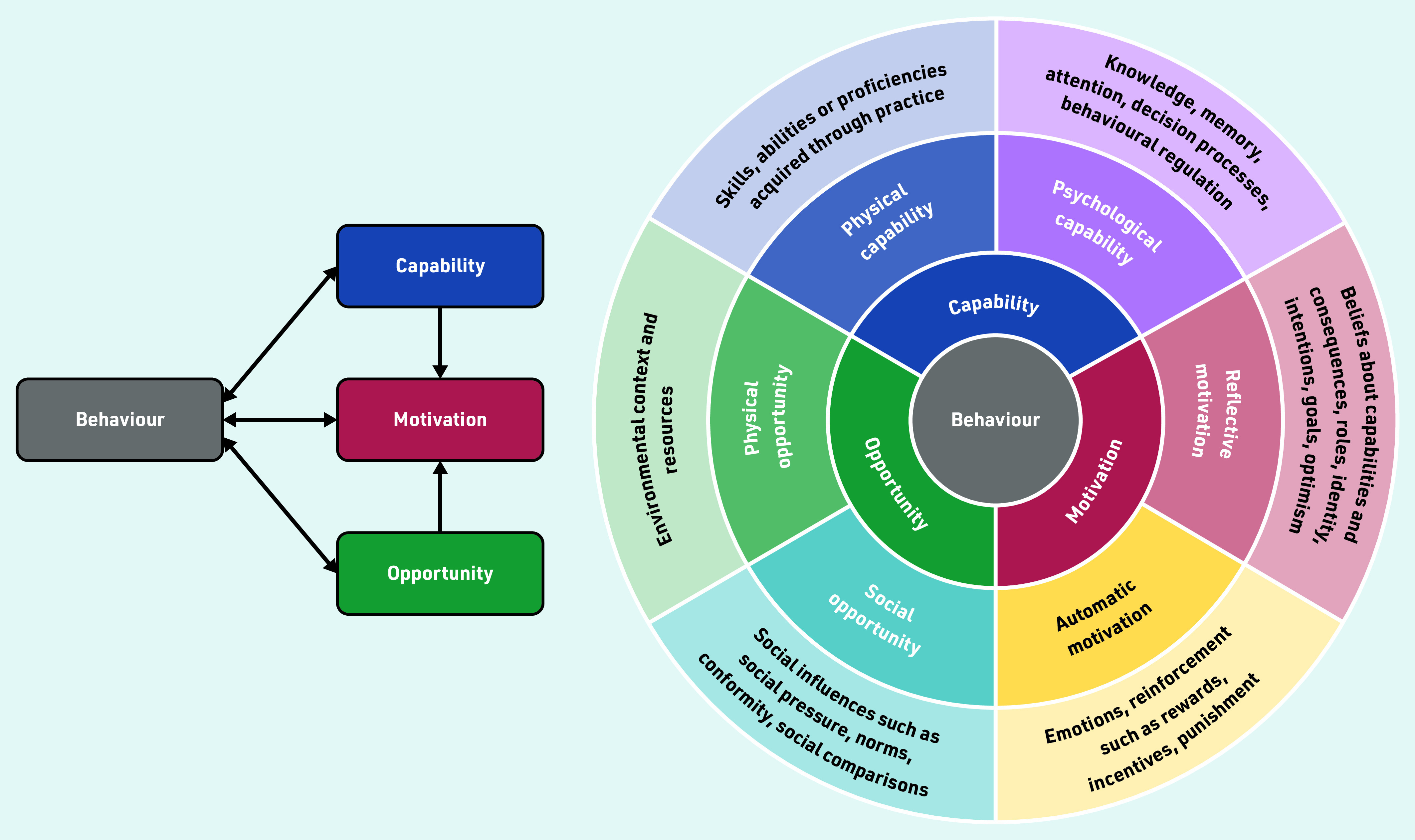
*Based on the COM-B model.^13^ © 2011 Michie S,* et al*; licensee BioMed Central Ltd. Reproduced under the terms of the Creative Commons Attribution License (http://creativecommons.org/licenses/by/2.0).*

The theoretical framework used was the established COM-B behaviour change model. This model was used because it identifies what component of behaviour, capability, opportunity, or motivation needs to be modified for an intervention, in this instance, PE, to be successful (Figure 1).^13^^,^^14^ COM-B has been used previously to understand variation in practice by GPs where social awkwardness is an issue: for instance, in relation to implementing testing for sexually transmitted infections.^15^ It also links to the behavioural change wheel that aids intervention development.^13^

## METHOD

Qualitative semi-structured telephone or face-to-face interviews were conducted with practising GPs in one health board in Scotland between March and June 2019. Fourteen NHS Grampian GP practices that had participated in a previous linked data analysis study involving patients diagnosed with a gynaecological cancer were approached (unpublished data, Pauline Williams, 2021). An introductory email containing a letter of invitation, study information sheet, and an expression of interest reply slip was sent to practice managers who cascaded it to the GPs. Reply slips were returned by post or email.

**Table:** How this fits in

Not carrying out a pelvic examination (PE) is associated with diagnostic delay in gynaecological cancers; however, PEs are often not carried out by GPs. This qualitative study reveals that GPs’ reasons for carrying out, or not carrying out, PEs in women with symptoms potentially indicating cancer are complex. GPs need to have the skills to do, and interpret the findings of, a PE; have the opportunity to carry out the examination in terms of time and availability of chaperones; and believe that it will influence the referral process.

Semi-structured interviews were conducted at a mutually convenient time and place, and face-to-face or by telephone according to interviewee preference. Written consent was obtained at face-to-face interviews, and recorded verbal consent was obtained from telephone participants. Participants could withdraw from the study at any stage either during or after the interview and any collected data was deleted. Interviews were conducted until data saturation was reached (three successive interviews with no new subthemes identified^16^).

Interviews followed a semi-structured topic guide (see Supplementary Appendix S1) based on the COM-B behaviour change model and developed iteratively as interviews progressed. All interviews were digitally recorded, transcribed verbatim, then imported into NVivo (version 12). A Framework approach^17^ was used. Analysis followed the steps of familiarisation, identifying a thematic framework, indexing, charting, and mapping/interpreting.

However, although COM-B was the framework, the generation of additional themes outside of the framework and overarching themes across elements of the framework were permitted. Indexing and charting were carried out on full transcripts by two authors. Mapping and interpretation were led by the first author, with contributions to this in meetings with all authors. Analysis was conducted iteratively alongside interviews.^17^

## RESULTS

Fifteen GPs (11 female) from nine practices were interviewed. Twelve worked in urban settings and three in rural. Five had advanced training in PE, as evidenced by holding a letter of competence in intrauterine techniques. Time since full GMC registration ranged from 9 to 35 years: two <10 years; three 11–20 years; eight 21–30 years; and two >30 years.

Themes in each of the COM-B components were identified (three within capability, two within opportunity, and three within motivation). In addition, two overarching themes of patient choice and practitioner gender were identified. In reporting the analysis, the authors recognise that some items could be described within multiple COM-B components. For instance, the delivery of much cervical screening and sexual and reproductive health by nurses in primary care reduces GPs opportunities to carry out PEs, and in turn leads to reduced capability through lack of continuing practice. Where this arises, factors have been described under one heading. Box 1 summarises the themes according to the COM-B framework.

**Box 1.** Summary of themes using the COM-B framework

**COM-B component**	**Theme**	**Theme details**
Capability	Learning	Early training
		Further training
	Continuing practice	Limited opportunities
		Loss of capability
	Different forms of capability	Procedural skill
		Confidence in interpretation

Opportunity	Chaperones	Patient preference
		Practical availability
	Clinical environment	Enhanced versus generic

Motivation	Doing the best for the individual	Prioritise patient over rules
	Doctors examine	Importance of checking
		Professional reputation
	GPs as pragmatists	Question added value of PE
		Referral as acceptable alternative

*PE = pelvic examination.*

### Capability

Participants acknowledged that capability was dependent on training opportunities, experience, and exposure in current clinical practice. There were three themes related to capability: learning, continuing practice, and different forms of capability.

#### Learning

Most interviewees regarded opportunities for learning PE in their undergraduate training as inadequate. Rotation through obstetrics and gynaecology as part of GP vocational training was considered a valuable component of learning although not all found it useful.

Other than one female interviewee, most did not recall any consolidation or further training of PE skills during GP registrar training; however, new approaches to GP registrar training with required competences listed in an e-portfolio were regarded as beneficial, likely to produce GPs with improved skills, as the following quote illustrates:

*‘So I think the new trainees coming out are much better than we were, because they’ve had to prove throughout their e-portfolio that they are doing intimate examinations* […] *so they’re usually examining and then the patient being re-examined by either a trainer or another colleague.’*

(GP4, female)

Interviewees were asked whether they thought continuing education in PE should be mandatory or voluntary and there was no consensus. In terms of what learning opportunities might be offered, suggestions included didactic lectures, attending gynaecological outpatient clinics, and use of pelvic models.

Interviewees recognised that capability required both training and continuing practice of PE.

#### Continuing practice

All GPs described the way that changes to primary care practice (particularly nurse-led cervical screening) had reduced their continuing practice of PEs:

*‘I think it might be very hard to get day to day experience with gynaecology and you could go weeks* […] *where you wouldn’t see anything.’*

(GP 15, female)

Male GPs were seen as less likely to have opportunities for continuing practice of PEs. Women were seen as more likely to choose a female GP for gynaecological problems and some felt that practice receptionists might triage women to female practitioners (GP or nurse) or those perceived as specialists. For example, practitioners trained to fit intrauterine devices were seen as more ‘specialised’ within the practice and may gain extra experience both by carrying out non-diagnostic PEs as part of device fitting and also by more general awareness of their expertise:

*‘You’ll have one person in the practice who does coils, so that even decreases skill sets for other female GPs, never mind the male GPs.’*

(GP 14, male)

There was consensus that higher levels of exposure contributed to higher levels of confidence in examination skills and capability, and was determined partly by the sex of the GP, as male GPs were less likely to carry out PEs. The requirement for chaperones (see later section) was perceived to be more problematic for male GPs and patients were perceived to self-select female GPs when presenting with gynaecological symptoms, as in UK primary care, patients can select their clinician. Those certified to fit intrauterine devices felt more skilled and confident because of increased non-diagnostic exposure, but this had an impact on other GPs’ exposure levels.

#### Different forms of capability

Perceptions of capability varied across the different elements of PE. Visualisation of the cervix was considered an essential part of the examination by all interviewees, although one male GP stated using a speculum would ‘cause him alarm’. However, several interviewees were concerned that they had insufficient capability to accurately identify cervical abnormalities. Bimanual examination was considered more difficult and subjective in interpretation. Its diagnostic value was questioned with concern about both false-negative and false-positive examinations. Most felt that overt changes would be correctly identified, but subtle changes were more likely to be misinterpreted or missed. No one mentioned examination of the vulva, the third component of PE.

In summary, capability in PE was seen as requiring adequate training and learning, continuing practice, and the ability not just to carry out examination but to interpret it accurately.

### Opportunity

Two themes related specifically to opportunity: chaperones and clinical environment.

#### Chaperones

Beliefs about the use of chaperones varied between GPs as did their actual use of chaperones; however, similar beliefs did not always result in the same behaviour.

All GPs felt the need for chaperones was an important obstacle to opportunity. All were aware of guidance from the Royal College of General Practitioners and the GMC. Although most GPs routinely offered chaperones, some did not. Although the offer of a chaperone was made, some felt the guidance did not benefit patients and were happy to carry out the examination without one:

*‘It* [using a chaperone] *makes the whole thing less natural, and more awkward* […] *it’s embarrassing enough for them to have me looking at their down belows, without somebody else.’*

(GP7, female)

Some female participants did not always offer a chaperone. Many said they had never had a patient request a chaperone:

*‘I’d say, are you okay if I examine you, and if they say yes, and they jump up on the bed, then that’s good enough for me.’*

(GP4, female)

Clear sex differences were observed regarding the belief that chaperones were essential, but one male GP indicated a situation where they would not always use a chaperone:

*‘I’ll do it on a post-menopausal lady* […] *or I’ll say if you’ve had your kids, and you’re comfortable with me doing it, and quite often they’ll say yes* […] *I’m exposing myself to some risk, but I think it’s pretty low, but anybody my age or younger certainly not.’*

(GP13, male)

This behaviour was driven by assumptions the GP had about patients’ beliefs:

*‘Elderly ladies* […] *I could be wrong, but they say this is just a procedure that needs to be done, I’m sure younger women do, but I do it, to protect myself, rather than them.’*

(GP13, male)

This need to protect themselves from potential litigation by offering a chaperone was also expressed by female GPs, despite some feeling that offering a chaperone was a tick-box exercise:

*‘I think that it’s one of these tick box sentences, that feels like, it feels so stupid to ask it* […] *do you want someone come in with you, they* [the patient] *look at you, like, what, what?’*

(GP5, female)

Female GPs also acknowledged not using a chaperone contradicted the guidance, but it was normal practice for several participants. This decision was influenced by familiarity with or age of the patient. The majority of those who offered a chaperone did not expect the patient to want one. Some GPs who routinely did not use a chaperone would suggest a chaperone if the patient made them feel uncomfortable, with GPs of both sexes acknowledging their vulnerability when carrying out PEs:

*‘I do offer the patient one, but if they don’t wish one* […] *I should get one anyway, for my own defence* […] *because I often know the patients quite well, I, I chose to take that risk.’*

(GP 8, female)

The guidance states chaperones should preferably be a health professional and familiar with PE procedures, however, participants commented that practice staff with these attributes are often engaged in their own clinical practice and not free to chaperone. One GP’s practice had trained reception staff to be chaperones to increase availability. However, for some practices even larger obstacles were present:

*‘We have a, a satellite surgery in* [location] *where it’s just us, you know, there’s no one, no admin or anybody.’*

(GP15, female)

Only one GP explicitly described the value of chaperones for those patients who were especially nervous about the examination.

#### Clinical environment

Participants described a number of issues relating to the clinical environment that either facilitated or obstructed the conduct of PEs. These included time and equipment such as adjustable examination couches. Interviewees gave examples of where these were absent. Routine consultations of 10 min were seen as insufficient for an adequate history and examination when compounded by the need to find a chaperone with no notice and ensure sufficient dignity for the patient. In contrast, designated appointments with appropriate time and equipment, especially where the practitioner had been selected for their expertise (for instance, by referral within the practice between clinicians) were seen as facilitating PEs. The issue of in-practice referral, especially from a male GP to female was discussed by several participants and was generally felt to be appropriate.

### Motivation

Interviewees described a range of motivations towards or against PEs that were independent of either capability or opportunity. Importantly all related to providing high-quality care, but this was defined in different ways. These are categorised broadly as three themes: ‘doing what is best for the individual’, ‘doctors examine’, and ‘GPs as pragmatists’.

#### Doing what is best for the individual

Some GPs made it clear that there were few hard and fast rules about when PEs should be carried out and that the highest priority in clinical decisions was the patient:

*‘We should be tailoring the investigation to the individual, with respect for their dignity, and asking the question, what will this investigation give me?’*

(GP3, female)

*‘It’s an intimate examination, it should only be done if it’s going to add to your, to your diagnosis, or management.’*

(GP8, female)

However, some GPs assumed that patients did not want to be examined because of embarrassment. They also assumed that patients would not wish to be examined by students, resulting in reduced learning opportunities.

#### Doctors should examine

Some participants emphasised the importance of clinical examination as something that doctors do. Thus, although the intimacy of PE should be acknowledged, this should not prevent it:

*‘I think it would be embarrassing to miss something for the want of, of their embarrassment or our discomfort at having to do it* […] *because if we miss that then, you know, they’re on a completely different path.’*

(GP6, female)

Some participants acknowledged that lack of examination could lead to misattribution of symptoms:

*‘I guess even post-menopausal bleeding for query endometrial* [cancer]*, usually I would examine them, just to make sure if there was anything local.’*

(GP8, female)

For some, not carrying out examination before referral, even if it was unlikely to add clinical value, was seen as unprofessional:

*‘You wouldn’t put* [refer] *someone to cardiology, if you hadn’t listened to their chest, they would laugh at you.’*

(GP2, female)

#### GPs as pragmatists

Some GPs explicitly questioned the diagnostic value of PE by a non-specialist. For instance, several argued that imaging offered more clinical yield than clinical examination and that they would refer for ultrasound scanning rather than use clinical examination for diagnosis, especially for a patient with post-menopausal bleeding.

For many women presenting with symptoms that might be indicative of gynaecological cancer, some GPs argued that a decision to refer could be made on history alone. Once ‘red flag’ features had been identified in the history, examination findings would not positively have an impact on triaging of the referral by the specialist, or the patient’s diagnostic journey. One GP argued that although a rectal examination was clinically useful for a suspected rectal cancer, a PE was less useful for at least some types of suspected gynaecological cancer:

*‘In the past couple of months, two women with post-menopausal bleeding, I didn’t even bother to examine them* […] *they need* [to be] *referred* […] *I’m not going to find anything of value* […] *I can see the utility of the, the rectal exam, in you know, for rectal cancer, because if you feel the tumour* […] *that’s going to potentially change the initial management.’*

(GP14, male)

This argument was often seen in GPs who described low capability and opportunity (and thus, for whom the probability of a well conducted and interpreted examination was likely to be lower).

Although GPs recognised clinical guidelines for urgent referral of patients with suspected cancer they described their limitations, especially when advice changed:

*‘I just have a gut feeling there’s something not right, they don’t necessarily fit a guideline, but they need* [to be] *referred.’*

(GP2, female)

Pragmatic GPs also saw limitations to the value of providing extra information at the time of referral:

*‘My referral letters tend to be quite short and to the point, so maybe mine are read more, but I know I’ve signed letters for some people who just write screeds of stuff, and think what’s the point, because who’s going to read this at the end?’*

(GP7, female)

### Integrating findings

While the analysis has been presented by deconstructing interviews into the COM-B framework, it is clear that a number of different factors influence GPs’ approach to carrying out PEs. These are presented in Box 2, which summarises the findings in a set of conditional statements, each of which makes PEs more or less likely in the GP consultation. This has value in two ways: increasing understanding and planning service innovation. In terms of understanding, it makes it apparent why factors (such as male sex of the GP) may have effects at multiple levels of this integrated model. In terms of service innovation, it suggests that action at a single level is unlikely to have a substantial impact without addressing others.

**Box 2.** Summary of conditions that make GP PE more or less likely

	**Conditions**	**PE in the GP consultation more likely**	**PE in the GP consultation less likely**
**Capability**	GPs who …	are well trained in PE	have not had detailed training in PE
		have a substantial PE caseload to maintain confidence in their skills	have a limited PE caseload and less confidence in their skills

**Opportunity**	GPs who practice in …	a clinical environment which has been enhanced to provide PE	a generic clinical environment
	… where chaperone needs …	are easily met	are at best hard to meet
	… and where patients with gynaecological symptoms …	choose, or are directed, to see them because of their expertise	have little choice in who they see

**Motivation**	GPs who believe that by carrying out PE …	they commonly add important value to the clinical process before or at the time of referral	they commonly add no value to a decision to refer either for imaging or specialist assessment

**Behaviour**	Outcome	GP is more likely to offer and conduct PE before referral	GP is less likely to offer or conduct PE, but, instead to either bypass PE (by direct referral) or to arrange for PE by a practice colleague

*PE = pelvic examination.*

## DISCUSSION

### Summary

The reasons why GPs do, or do not, use PEs in assessing gynaecological symptoms that might indicate cancer are complex. The COM-B model provides a way to understand GPs behaviour that shows how contrasting views about the value and practice of examination depend on factors relating to all three components: capability, opportunity, and motivation. A framework that integrates these findings allows these factors to be considered together and could be used both in understanding individual decisions and in planning training or innovation in the way services are delivered.

### Strengths and limitations

To the authors’ knowledge, this is the first study of its kind that explores GPs’ perceptions of PE using the COM-B behaviour change model as the analytical framework. Analysis was carried out by all researchers: three GPs, a pharmacist, and a consultant gynaecologist. Preconceptions of researchers can be a challenge, but the multidisciplinary approach helped to mitigate this.

There were, however, some limitations. Study participants knew the interviewer (the first author) was a GP, potentially influencing responses. As with all exploratory studies the results are not intended to be generalisable, but interviewee selection should aim to recruit a range of characteristics likely to affect views and experiences. Undertaking the study in one health board in Scotland and the self-selection of GPs agreeing to be interviewed may mean there are other views that have not been identified.

### Comparison with existing literature

Existing literature is limited. Paluska and D’Amico presented data in 2000 confirming doctors are more comfortable examining patients of their own sex.^18^ They assumed this was because male doctors felt patients would be embarrassed; however, questioning patients has revealed that despite feeling nervous about PEs, patients regard it as necessary.^19^ Although patients report being exposed during the examination, they also describe how clinicians can influence this discomfort. Good communication during the examination, a trusted relationship with the clinician, and a comfortable environment can reduce patient distress.^19^^–^^21^ Clinician discomfort can increase patient’s distress.^21^ Clinician discomfort can also influence their use of chaperones. Reflecting the findings in the current study, discordance between clinical practice and guideline recommendations has been observed previously and has been quantified; whereas 72% of clinician’s thought patients should be offered a chaperone, only 27% of the study population used chaperones.^22^ Another study suggested that guidance on the use of chaperones should be flexible, taking into consideration the relationship between patients and their doctors while considering practice staffing and space;^23^ this pragmatic approach was observed in this study.

Chaperone use was acknowledged as a barrier in terms of resource. But there was also recognition of clinical vulnerability and potential litigation while acknowledging that patients generally did not want chaperones. The use of chaperones to alleviate feelings of vulnerability to false allegations or to sexualisation of the examination by the patient has been reported by Hine and Smith.^24^

### Implications for research and practice

The identification in the current study of important factors in all three elements of the COM-B framework indicates that interventions to change practice either through training or service delivery innovations need to address multiple components. As suggested in Box 2, changing one element without addressing others is likely to have limited effect. It is therefore important to consider all three components.

Increasing capability could involve more skills training with the use of pelvic mannequins; however, these are not anatomically representative of many women and do not allow the development of communication skills that are key in reducing patient concerns. An alternative could be gynaecological teaching associates (GTA). A GTA is a non-doctor woman who has been trained to teach PE using their own bodies.^25^ They are able to develop a patient-centred approach while removing the homogeneous depictions of women in mannequins. Their value in improving medical student education is well established,^26^ but it is not clear how effective they are for maintaining skills after initial training.

Several practices described ways of delivering care that addressed opportunity, for instance, supporting patient choice of, or explicitly directing patients to, GPs with greater experience. Further consideration is required to determine how patients access those confident GPs at the earliest possible opportunity. Ten-minute consultations and difficulties in locating appropriate chaperones clearly reduce opportunity. Increased length of consultation has also been highlighted by the Royal College of Obstetricians and Gynaecologists report, *‘Better for Women’*, which proposes 15-min GP consultations.^27^ Additionally, primary care has changed its consultation model because of the COVID-19 pandemic. The authors do not know in what way this has affected how clinicians carry out PEs.

Motivation factors about PE are clearly complex. GPs remain unsure about how valuable examination findings are to a good gynaecological referral. This could be addressed in further database studies, but as a starting point, gynaecologists should be asked whether they value PEs carried out in primary care. More research is also needed to understand how much GPs assumptions about what women prefer match to the reality.

The unheard voice in this research is of women themselves. While the authors have knowledge of patients’ views on PE, the authors do not know how this influences their help-seeking behaviour. Delayed help seeking is associated with poorer outcomes.^28^ Research is needed to understand how PE influences patients’ help-seeking behaviour.

In conclusion, GPs’ reasons for using, or not using, PE in women with symptoms potentially indicating cancer are complex. The COM-B framework provides a way of understanding this complexity. Interventions to increase the use of PE, and critics of its non-use, need to consider these multiple factors.
